# Supplementary material for: Comparative genomics provides new insights into the diversity, physiology, and sexuality of the only industrially exploited tremellomycete: Phaffia rhodozyma
Source: BMC Genomics. 2016 Nov 9;17:901. doi: 10.1186/s12864-016-3244-7 (PMC5103461; doi:10.1186/s12864-016-3244-7)
Supplement: Additional file 6: — List of orphan genes with links to PFAM (related to Additional file 1: Table S1). (ZIP 1428 kb) [file 12864_2016_3244_MOESM6_ESM.zip › PFAM_UNIQUE/G02106_P.html]

#                                                                            --- full sequence --- -------------- this domain -------------   hmm coord   ali coord   env coord
  
# target name        accession   tlen query name           accession   qlen   E-value  score  bias   #  of  c-Evalue  i-Evalue  score  bias  from    to  from    to  from    to  acc description of target
  
#------------------- ---------- ----- -------------------- ---------- ----- --------- ------ ----- --- --- --------- --------- ------ ----- ----- ----- ----- ----- ----- ----- ---- ---------------------
  
G02106\_P             -            556 GlyL\_C               PF12524.3     65     0.057   12.0   5.2   1   2   3.3e-05     0.099   11.3   2.5    20    57   179   217   174   225 0.84 - dsDNA virus glycoprotein L C terminal  
G02106\_P             -            556 GlyL\_C               PF12524.3     65     0.057   12.0   5.2   2   2      0.38   1.1e+03   -1.8   0.0    30    44   236   250   218   257 0.73 - dsDNA virus glycoprotein L C terminal  
G02106\_P             -            556 HHV6-IE              PF03753.8    993       1.7    4.7  16.8   1   1   0.00076       2.3    4.3  16.8   506   696    67   263    44   289 0.68 - Human herpesvirus 6 immediate early protein  
G02106\_P             -            556 SSB                  PF00436.20   104    0.0058   15.2   0.0   1   1   3.5e-06     0.021   13.4   0.0     4    66   442   500   440   521 0.80 - Single-strand binding protein family
